# Exome resequencing identifies novel NPHP genes, implicating DNA damage response signaling in the pathogenesis of ciliopathies

**DOI:** 10.1186/2046-2530-1-S1-O2

**Published:** 2012-11-16

**Authors:** F Hildebrandt

**Affiliations:** 1 Howard Hughes Medical Institute and University of Michigan, Ann Arbor, MI, USA

## 

Nephronophthisis-related ciliopathies (NPHP-RC) are recessive multisystem-disorders that affect kidney, retina, liver and cerebellum either by prenatal-onset dysplasia or by childhood-onset degeneration and fibrosis. Identification of 10 disease-causing genes (*NPHP1-NPHP10*) revealed that their products are located at primary cilia and centrosomes. However, the proximal disease mechanisms remain poorly understood. We identified by whole exome resequencing, mutations that affect the centrosomal proteins FAN1, MRE11, ZNF423, and CEP164, as novel causes of NPHP-RC. Surprisingly, these ciliopathy genes serve functions within the DNA damage response (DDR) pathway (Chaki & Airik et al Cell, in press). Specifically, i) We identify a homozygous truncating mutation (W707X) of *FAN1* in 2 siblings with karyomegalic interstitial nephritis (KIN), which is histologically indistinguishable from NPHP, except for the feature of karyomegaly. FAN1 is essential for the DNA interstrand crosslink (ICL) repair pathway of DDR signaling. The KIN renal phenotype is mimicked by ICL-causing genotoxins including busulfan and ochratoxin A. ii) We identify in siblings with cerebellar vermis hypoplasia (CVH) a homozygous truncation mutation of *MRE11* (R633X), which is an essential component of the ATM-Chk2 pathway of DDR, where MRE11 recruits ATM to sites of DNA double-strand breaks. iii) We identify in 2 siblings with nephronophthisis and CVH a homozygous missense mutation (P913L) in the zinc finger-encoding gene *ZNF423*. Ablation of the homologous gene (*Zfp423*) was shown to generate CVH in mice due to defects of cerebellar progenitor cell differentiation. Interestingly, ZNF423 interacts with the DNA ds-break sensor PARP1, which recruits MRE11 (MRN) and ATM to sites of DNA damage. iv) ATM, in turn, is activated by MRE11 (MRN) and the ‘TIP60 complex’. And we demonstrate colocalization to TIP60-positive (and SC35-positive) nuclear foci or protein-protein interactions for the following products of genes mutated in NPHP-RC: SDCCAG8/NPHP10, ZNF423, CEP164, OFD1, RUVBL1, RUVBL2, NPHP5, NPHP1, and ATXN10. OFD1, RUVBL1, RUVBL2, are known to play a role in DDR. vi) Finally, in 4 different families with NPHP-RC we identify recessive mutations of *CEP164* as a novel cause of NPHP-RC. CEP164 acts in the ATR-Chk1-related arm of DDR, where it is necessary for ATR-dependent Chk1 activation upon induced replication stress (Sivasubramaniam 2008). We suggest a new pathogenic working hypothesis for certain forms of NPHP-RC proposing the following cascade of events: i) defects of DDR → lack of Chk1 (Chk2) activation → inadequate G2/M cell cycle arrest. This would lead in high proliferation states (high replication stress) during morphogenesis to dysplastic phenotypes (Meckel syndrome) and in low proliferation states (low replication stress) during tissue maintenance and repair to tissue degeneration and fibrosis (nephronophthisis), perhaps due to loss of adult progenitor cells.

